# An Uncommon Cause of Cervical Pain in a Child: Osteoblastoma of the Cervical Spine

**DOI:** 10.7759/cureus.100714

**Published:** 2026-01-03

**Authors:** An T Hoang, Cerys Arnold, Kathryn A Szymanski, Dane Van Tassel

**Affiliations:** 1 Radiology, Creighton University School of Medicine, Phoenix, USA; 2 Radiology, University of California Los Angeles David Geffen School of Medicine, Los Angeles, USA; 3 Radiology, Phoenix Children's Hospital, Phoenix, USA

**Keywords:** cervical osteoblastoma, pediatric back pain, pediatric msk radiology, pediatric radiology, radiology

## Abstract

Osteoblastoma in children is a rare diagnosis of primary bone tumors. This case report describes a C5 osteoblastoma in a 12-year-old male patient with upper back and neck pain. While initial radiographs were unrevealing, CT and MRI showed an impinging left C5 pedicle mass, with neural foramina narrowing. Pathology reports confirmed an osteoblastic lesion without malignant characteristics. Infrequent occurrence of osteoblastoma and potentially inconclusive radiographs may lead to diagnostic delays. Thorough evaluation of back pain in pediatric patients is critical in promptly detecting osteoblastoma and other neoplasms.

## Introduction

Osteoblastoma is a rare, benign bone-forming neoplasm, with an incidence of 1% of all primary tumors. It typically arises from posterior spinal elements and may exert compressive effects on adjacent nerve roots. It often affects the spine (30-40% of cases), long bones, and, less commonly, the jaw [1-2]. It is characterized by the abnormal formation of osteoid and immature bone, leading to localized pain that may aggravate at night [3-5]. While osteoblastomas can occur at any age, they are most commonly diagnosed in children and young adults [1], though few pediatric cervical cases are published in the literature. Osteoblastomas can be confused with osteoid osteoma histologically, with increased osteoid tissue formation surrounded by vascular fibrous stroma and perilesional sclerosis. Osteoid osteoma, a small (usually <2 cm) common benign pediatric tumor, is observed mostly in long bones with limited expansive growth and nocturnal pain that is relieved by nonsteroidal anti-inflammatory drugs (NSAIDs). In contrast, osteoblastoma is larger (2-15 cm), most often at the axial skeleton, with pain that is usually not relieved by NSAIDs [6-8]. Nonspecific findings, such as dull and localized pain, may lead to delayed diagnosis. While the tumor is generally slow growing, its increasing size, especially in the cervical spine, puts patients at a high risk for severe pain, aggressive skeletal defect (e.g., scoliosis), and neurological symptoms due to nerve root compression (e.g., radiculopathy, muscle weakness, paraplegia, bowel and bladder dysfunction) [8-9]. Additionally, research has found a high recurrence rate with potential malignant transformation. The diagnosis of osteoblastoma begins with radiographs, which may be hard to differentiate from osteoid osteoma, requiring additional CT imaging. CT scans complement radiographs in clarifying the internal matrix and further characterizing the size and extent of disruption. MRI scans identify the extent of peripheral tissue edema and may localize neuroforaminal compromise, guiding treatment for more aggressive osteoblastomas. Treatment of osteoblastoma is surgery, with en bloc resection or curettage, and is case-dependent (size, location, concern for malignancy) though en bloc resection has resulted in lower local recurrence with higher complication risks due to its invasiveness as found in Zhang et al. [10]. Frequent imaging monitoring is important due to the high recurrence rate. 

This case report describes a 12-year-old male patient who presented with persistent upper back and neck pain. Initial imaging was inconclusive, but further investigation with MRI and CT ultimately led to the diagnosis of osteoblastoma, a rare benign bone tumor of the cervical spine. This case highlights the importance of thorough diagnostic evaluation and consideration of osteoblastoma in a broad differential when dealing with neck or back pain in pediatric patients. 

## Case presentation

A previously healthy 12-year-old male patient presented to the emergency department with a chief complaint of intermittent left upper back and neck pain that began three months ago; back pain was localized midline to the scapula. He described the pain as worse in the morning and aggravated by movement, resulting in restricted physical activity and range of motion. He denied radicular pain as well as any other motor or sensory deficits. He reported using ibuprofen for pain relief, though it was unclear whether symptoms improved with medication. He also described three months of gradual weight loss and several weeks of intermittent night sweats and fatigue. On presentation, he was tachycardic (138 beats per minute) and febrile with a temperature of 39.1°C. 

**Figure 1:**
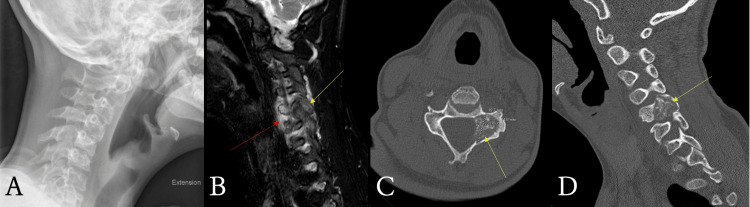
Imaging studies of the cervical spine including (A) radiograph left lateral view without obvious abnormality, (B) MRI sagittal view with T2 hypointense enhancing mass in the C5 pedicle (yellow arrow) with associated marrow edema of the C5 vertebral body and surrounding paraspinal soft tissue edema (red arrow), and CT without contrast (C) axial view measuring an osseous mass of the C5 left pedicle (yellow arrow) at 19.7 mm craniocaudally x 14.9 mm AP and (D) sagittal view showing a mass of the C5 pedicle (yellow arrow) with severe C5-C6 foraminal narrowing AP: anteroposterior

Physical examination was notable for cervical spine and left paraspinal tenderness to palpation with bilateral limitation in neck range of motion; the remainder of the exam was unremarkable. Laboratory evaluation revealed a normal complete blood count and basic metabolic panel, aside from an elevated lactate dehydrogenase level. Same-day cervical spine radiography showed no acute abnormalities (Figure 1A), and he was referred to physical therapy while awaiting authorization and scheduling of an MRI. Supportive care instructions for presumed concurrent influenza infection were also provided based on reassuring laboratory findings and chest imaging. His influenza symptoms resolved shortly after discharge. 

Three months later, a cervical spine MRI with and without contrast revealed an enhancing osseous mass centered approximately in the left C5 pedicle with surrounding marrow and soft tissue edema and mass effect on the regional nerve roots (Figure 1B). A noncontrast CT scan of the spine was subsequently performed to obtain further detail. The CT scan revealed a 2.0 cm lesion in the left C5 pedicle (Figure 1C), along with severe C5-C6 neural foraminal narrowing, compressing the exiting left C6 nerve root (Figure 1D). This narrowed the differential diagnoses to osteoblastoma versus chondroid tumor. 

He underwent curettage of the C5 bone tumor and bone grafting two months after his MRI and CT scan with no complications. Pathology reports showed an osteoblastic lesion with disorganized deposition of osteoid, exhibiting varying degrees of mineralization. The intervening space contained numerous osteoblasts and scattered osteoclasts, with no signs of increased mitosis, hypercellularity, or atypia. These findings, in conjunction with the imaging results, strongly suggested a diagnosis of osteoblastoma. 

He was discharged on postoperative day 3 with multimodal pain management, a cervical spine brace, and a rehabilitation plan. At his six-month follow-up, cervical radiographs demonstrated new bone formation at C5 without evidence of lytic lesions, although he reported neck weakness that was gradually improving. He was subsequently cleared for full return to physical activity and instructed to discontinue the cervical brace. 

At his seven-month follow-up, radiographs showed increased bone maturation, evident by an increased intensity, compared with prior imaging, and no signs of recurrence. At that time, he regained full cervical range of motion and denied any motor, sensory, or pain symptoms. 

## Discussion

Osteoblastoma is a rare, benign bone tumor, most commonly occurring in the spine. Its presentation in the cervical spine accounts for only 20% of cases, and in a pediatric patient, it is even less frequent [1]. Symptoms are often nonspecific and may include localized progressive back pain, which may be aggravated by activity or at night [11-12]. The absence of significant findings on initial imaging and nonspecific symptoms can make early diagnosis challenging. 

In pediatric patients presenting with neck and upper back pain, many differential diagnoses may be considered, including benign and malignant causes. Though pain related to muscular fatigue and strain is common, other diagnoses must be considered, particularly when red flag symptoms are present. For example, osteoid osteoma and, less commonly, lipomas may be localized in the same region and present similarly to our patient [13-15]. Although an osteoid osteoma was initially suspected, the patient’s lesion exceeded the typical osteoid osteoma size of <2 cm, making osteoblastoma a more likely diagnosis [6-8]. Additionally, neoplastic processes, such as lymphoma, rhabdomyosarcoma, or neuroblastoma, can also present with neck masses and associated pain, though these are often accompanied by systemic signs like fever, weight loss, or unexplained fatigue, several of which were present in this patient, further complicating the diagnostic picture [2,3,11]. However, the reassuring initial radiograph, largely unremarkable laboratory studies, and subsequent resolution of systemic symptoms at follow-up favored a benign lesion. Thorough history-taking, appropriate laboratory evaluation, and advanced imaging are essential to refine the differential diagnosis and guide timely management. 

In spine/posterior-element lesions, radiographs may be normal or nonspecific, such as in our case. The thorough evaluation with MRI and CT imaging allowed for a more detailed evaluation of the osseous structures, provided clarity in identifying and localizing the mass, and provided information regarding soft tissue involvement. In literature, CT proves to be the best at localizing osteoblastoma, though MRI is superior in identifying related secondary nerve compressions [9,12,16]. On CT, osteoblastomas typically present with a lytic mass that may show scattered calcification or be enclosed in sclerosis [9]. Due to its overlapping imaging features, osteoblastoma of the cervical spine in a pediatric population may be challenging to differentiate from other pathologies, requiring more detailed imaging. 

Treatment typically involves surgical resection, and additional management may vary based on clinical situation and location. Management options include minimally invasive techniques, such as radiofrequency ablation (most commonly used), cryoablation, and laser ablation, for well-localized lesions away from critical neurovascular structures [17-18]. Surgical management ranges from intralesional excision or curettage for less aggressive lesions to marginal or en bloc resection for more aggressive disease, with spinal fusion or reconstruction when stability is compromised [16,19,20]. Preoperative embolization can reduce blood loss in highly vascular tumors, and adjuvant radiotherapy is considered when complete resection is not possible. Jiang et al. reported that nine of eleven patients with osteoblatomas had recurrence with curettage followed by radiotherapy, and no recurrence in patients who underwent invasive en bloc resection and minimally invasive vertebrectomy [19]. A meta-analysis, performed by Zhang et al., compared en bloc resection with debulking surgical technique (e.g., curettage) involving 1135 patients, showed lower recurrence rate (p < 0.00001), lower postoperative metastasis rate (p = 0.002) and lower mortality rate ( p < 0.00001); of note, data included surgical treatments and outcomes that were not specific to osteoblastomas and were inclusive of other spine and bone tumors [10]. While minimally invasive approaches are on the rise, further research is needed to determine outcomes. Treatment should be individualized based on tumor location, aggressiveness, and patient age. 

This case highlights the critical role of primary care physicians and pediatricians in considering a wide variety of differential diagnoses when a child presents with a neck or back mass and pain. Equally important is the recognition of red-flag symptoms (e.g., weight loss, night sweats, and fatigue), as demonstrated in this patient, which should prompt consideration of more aggressive malignancies until proven otherwise. CT and MRI should be used to further investigate inconclusive plain radiographs in pediatric populations with complaints of progressive cervical spinal pain. Osteoblastoma, though rare in the pediatric cervical spine, should remain in the differential when a benign bone tumor is suspected, particularly when the clinical presentation and imaging findings align with this diagnosis. 

## Conclusions

Osteoblastoma should be considered in the differential diagnosis for pediatric patients presenting with persistent neck and upper back pain. Due to its rare occurrence and nonspecific symptoms, osteoblastoma can be difficult to diagnose. Delays in management may cause damage to surrounding structures if left untreated. Early diagnosis is crucial, and imaging modalities such as MRI and CT play a vital role in revealing its distinctive features, facilitating timely intervention. 
